# Impact of COVID-19 on primary care contacts with children and young people in England: longitudinal trends study 2015–2020

**DOI:** 10.3399/BJGP.2021.0643

**Published:** 2022-06-07

**Authors:** Kimberley A Foley, Edward J Maile, Alex Bottle, Francesca K Neale, Russell M Viner, Simon E Kenny, Azeem Majeed, Dougal S Hargreaves, Sonia Saxena

**Affiliations:** Department of Primary Care and Public Health, Imperial College London, London.; Department of Primary Care and Public Health, Imperial College London, London.; Department of Primary Care and Public Health, Imperial College London, London.; Department of Primary Care and Public Health, Imperial College London, London.; Population, Policy & Practice Research Programme, UCL Great Ormond Street Institute of Child Health Population Policy and Practice, London.; Alder Hey Children’s NHS Foundation Trust; professor, Department of Women’s and Children’s Health, University of Liverpool, Liverpool; national clinical director, NHS England and Improvement, London.; Department of Primary Care and Public Health, Imperial College London, London.; Mohn Centre for Children’s Health and Wellbeing, Imperial College London, London.; Department of Primary Care and Public Health, Imperial College London, London.

**Keywords:** adolescent, child, COVID-19, general practice, primary health care

## Abstract

**Background:**

The NHS response to COVID-19 altered provision and access to primary care.

**Aim:**

To examine the impact of COVID-19 on GP contacts with children and young people (CYP) in England.

**Design and setting:**

A longitudinal trends analysis was undertaken using electronic health records from the Clinical Practice Research Datalink (CPRD) Aurum database.

**Method:**

All CYP aged <25 years registered with a GP in the CPRD Aurum database were included. The number of total, remote, and face-to-face contacts during the first UK lockdown (March to June 2020) were compared with the mean contacts for comparable weeks from 2015 to 2019.

**Results:**

In total, 47 607 765 GP contacts with 4 307 120 CYP were included. GP contacts fell 41% during the first lockdown compared with previous years. Children aged 1–14 years had greater falls in total contacts (≥50%) compared with infants and those aged 15–24 years. Face-to-face contacts fell by 88%, with the greatest falls occurring among children aged 1–14 years (>90%). Remote contacts more than doubled, increasing most in infants (over 2.5-fold). Total contacts for respiratory illnesses fell by 74% whereas contacts for common non-transmissible conditions shifted largely to remote contacts, mitigating the total fall (31%).

**Conclusion:**

During the COVID-19 pandemic, CYP’s contact with GPs fell, particularly for face-to-face assessments. This may be explained by a lower incidence of respiratory illnesses because of fewer social contacts and changing health-seeking behaviour. The large shift to remote contacts mitigated total falls in contacts for some age groups and for common non-transmissible conditions.

## INTRODUCTION

During the COVID-19 pandemic, health systems globally shifted towards treating COVID-19 infection in adults and minimising use of health services for other patients, including children and young people (CYP), who were less susceptible to severe COVID-19.^1^ On 5 March 2020, the NHS recommended remote triaging before any face-to-face contact to reduce infection risk.^2^ The UK Government announced a nationwide lockdown from 23 March 2020, and the public was advised to stay at home to limit transmission of COVID-19 and avoid strain on health resources.^3^ GPs were asked to prioritise consultations for urgent and serious conditions, and suspend routine appointments for planned or preventive care.^4^ During March 2020, the number of consultations in primary care decreased by a third,^5^ from about 6 million consultations to 4.2 million consultations a week. Face-to-face appointments were drastically reduced, while telephone appointments more than doubled.^5^ Reports of difficulty accessing GP appointments during the COVID-19 lockdowns led to concerns about patients’ unmet needs, which led to NHS England cautioning GPs to offer face-to-face appointments.^6^ However, GP leaders responded that general practice had complied with official guidance to offer predominantly remote services. GP surgeries remained open throughout the pandemic despite a dramatic increase in workload to adapt to the pandemic and manage COVID-19 infection.^7^

Children’s access to primary care is highly sensitive to health system shocks. The literature on primary care sensitive conditions has shown how rising pressures to manage a growing burden of adult chronic disease in primary care have squeezed children out of primary care and been associated with increases in children’s emergency department visits and hospital admissions for common non-transmissible conditions such as urinary tract infections, diabetes, epilepsy, and appendicitis.^8^^–^^11^ It became apparent early in the pandemic that CYP were less likely to become seriously unwell from COVID-19 infection.^1^ During the initial lockdowns, children aged <15 years, who are usually among the highest users of health services,^11^^,^^12^ had fewer emergency department visits compared with previous years.^13^ There was little evidence that these falls led to serious harms, an increase in mortality, or more severe illness presentations in CYP.^13^ However, it is not known how CYP’s consultation patterns changed in primary care, and whether the falls and changes in type of consulting reported for adults were similar for CYP.

**Table:** How this fits in

The COVID-19 pandemic response led to health system reorganisation globally, but its impact on children and young people’s (CYP’s) access to primary care is largely unknown. CYP’s health contacts with GPs fell by 41%, equivalent to 2.8 million fewer contacts in England, during the first COVID-19 pandemic lockdown from March to June 2020 compared with the previous 5 years. Face-to-face contacts with GPs fell by 88%, with a corresponding increase in remote contacts. The greatest falls in face-to-face contacts occurred among children aged 1–14 years (>90%). Remote contacts with infants and with young people aged 15–24 years more than doubled, mitigating some of the total falls in these age groups. GP contacts for respiratory illnesses fell 74% during lockdown compared with previous years, whereas there was less of a fall (31%) for contacts for common non-transmissible conditions (urinary tract infections, appendicitis, diabetes, and epilepsy).

This study therefore aimed to examine the impact of the COVID-19 pandemic on total, face-to-face, and remote GP contacts with CYP in England.

## METHOD

### Study population and data sources

A cross-sectional descriptive study was conducted using electronic health records from the Clinical Practice Research Datalink (CPRD) Aurum database. CPRD Aurum contains electronic health record data routinely collected from GP practices using EMIS clinical systems in England and Northern Ireland, covering over 13% of the population.^14^ It is generally representative both of the population in England and across its geographic regions in terms of age, sex, and deprivation status.^14^

The study population included all CYP aged <25 years who were registered with a GP practice in the CPRD Aurum database anytime during the study period (3 January 2015 to 30 October 2020). CYP were included if they had an ‘acceptable’ flag recorded in the database, indicating that their data met certain quality standards for key variables.^14^ Electronic health record data was included from 3 months before the formal registration start date with that practice. Any health records collected after de-registration and with young people aged ≥25 years were excluded. All GP contacts were assigned to one of six developmental age groups according to their age in years on the date of the health contact: <1 years, 1–4 years, 5–9 years, 10–14 years, 15–19 years, and 20–24 years.

### Outcomes

The main outcome was the total number of weekly contacts with a GP using codes for staff identity. Contacts with practice nurses and allied health practitioners, such as healthcare assistants, were excluded. Contacts included both ‘remote’ (telephone, video, or online) and ‘face-to-face’ consultations, and were defined in the CPRD Aurum database using administrative and clinical codes. This process is described in detail in Supplementary Box S1 and Supplementary Figure S1, and relevant code lists can be found in Supplementary Tables S1 and S2.

To describe the total healthcare activity, all face-to-face and remote contacts with GPs were included, even if there were multiple contacts with the same child or young person within a single day.

The secondary outcomes were weekly number of contacts with GPs for respiratory illnesses (upper respiratory tract infections, lower respiratory tract infections, and asthma) and primary care sensitive conditions (these are common non-transmissible conditions including urinary tract infections, diabetes, epilepsy, and appendicitis that are amenable to timely primary care but without which may result in potentially avoidable hospital admissions). The authors convened a clinician–researcher panel to develop and build code lists for each of these conditions using the CPRD Aurum code browser and existing lists from prior publications and code list repositories (see Supplementary Table S3).^15^^–^^25^

### Exposure definition

The first lockdown period in the UK was defined as 21 March to 5 June 2020, which corresponds to weeks 13 to 23 (inclusive) set by the Office for National Statistics. This time period was selected because of the timing of government restrictions and social distancing measures in place.^26^

### Data analysis

The number of contacts with GPs in each week from January 2015 to October 2020 was plotted. The percentage change in all outcomes was calculated by comparing the number of contacts during weeks 13 to 23 of 2020 with the mean number of contacts for weeks 13 to 23 from 2015 to 2019 to ensure more robust estimates of secular trends. All analyses were completed using Stata (version 17).

## RESULTS

**Figure 1.**
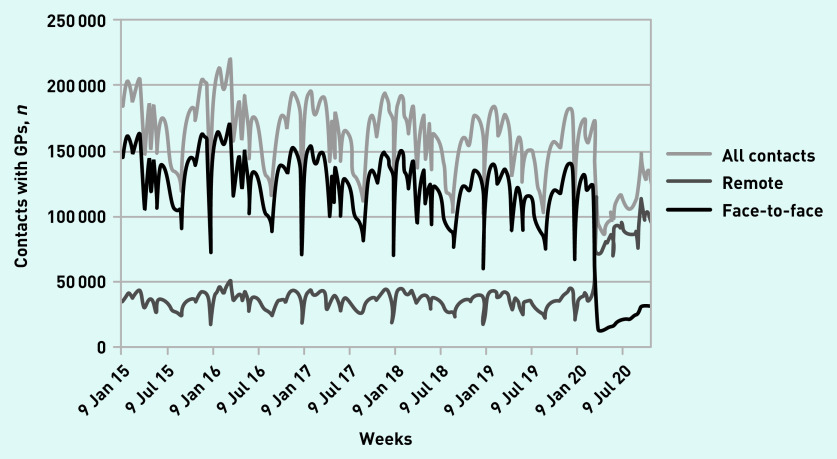
*The number of contacts with GPs in each week from January 2015 to October 2020.*

There were 47 607 765 GP contacts included in this study from January 2015 to October 2020. There were 3 927 298 CYP included at the start of the study, rising to 4 307 120 by the end of the study period. Total contacts with GPs for CYP fell by 40.7% (95% confidence interval [CI] = 35.8 to 45.1) during the first lockdown weeks in the UK (21 March to 5 June 2020) from an average of 1 752 874 over the same weeks (March to June) in 2015–2019 to 1 038 832 in March to June 2020 (Table 1). There were falls in total contacts for all age groups. After the initial lockdown eased in June 2020, total contacts with GPs rose, but by the week ending 23 October 2020 they remained 18% below the average for that week over the previous 4 years (Figure 1).

**Table 1.** Changes in the numbers of contacts with GPs by age group from March 21 2020 to June 5 2020 (week 13 to week 23) compared with the 5-year average during the same time period (week 13 to week 23) in 2015 to 2019

**Age group, years**	**Total number of contacts (2020)**	**Average number of total contacts (2015–2019)**	**Change in total number of contacts with GP, *n* (%; 95% CI)**	**Change in remote contact with GP, *n* (%; 95% CI)**	**Change in face-to-face contact with GP, *n* (%; 95% CI)**
<1	142 674	201 861	−59 187 (−29.3; 24.4 to 34.3)	+65 401 (+165.2; 148.4 to 182.2)	−124 588 (−76.8; 74.6 to 79.0)
1–4	169 846	357 496	−187 650 (−52.5; 47.4 to 57.1)	+65 169 (+80.1; 64.9 to 99.1)	−252 819 (−91.5; 90.5 to 92.4)
5–9	134 025	265 913	−131 888 (−49.6; 45.3 to 53.8)	+56 241 (+94.1; 79.4 to 111.0)	−188 128 (−91.2; 90.3 to 92.1)
10–14	111 090	229 903	−118 813 (−51.7; 46.7 to 56.2)	+45 089 (+90.0; 72.4 to 110.2)	−163 903 (−91.1; 90.0 to 92.1)
15–19	186 084	281 719	−95 635 (−33.9; 28.1 to 39.7)	+99 518 (+161.9; 141.1 to 183.5)	−195 153 (−88.6; 87.3 to 89.9)
20–24	295 113	415 982	−120 869 (−29.1; 24.3 to 33.8)	+155 881 (+155.6; 139.4 to 172.4)	−276 749 (−87.6; 86.5 to 88.7)
Total	1 038 832	1 752 874	−714 042 (−40.7; 35.8 to 45.1)	+487 298 (+124.2; 107.6 to 141.3)	−1 201 340 (−88.3; 87.2 to 89.4)

Face-to-face contacts fell by 1 201 340 (88.3%, 95% CI = 87.2 to 89.4) for all ages (Table 1), from an average of 1 360 490 in previous years to 159 150 in 2020 (Figure 1). The greatest falls in face-to-face contacts occurred among children aged 1–14 years (>90%), whereas face-to-face contacts with infants fell by 76.8% (95% CI = 74.6 to 79.0) (Table 1).

From March 2015 to June 2019, an average of 22% of the total weekly contacts with GPs were remote, compared with 85% of all GP contacts held remotely in March to June 2020 (Figure 1). Remote contacts more than doubled from an average of 392 384 in 2015–2019 to 879 682 in 2020 (Table 1). The week before the first lockdown, there was a large spike in remote contacts of 2.8 times (∼176%) compared with the average for that week in the previous 5 years (Figure 1). Infants, as well as young people aged >15 years, had >2.5 times more remote contacts during the lockdown compared with previous years, mitigating the overall drop in total contacts among these age groups (Table 1). Remote contacts also increased in children aged 1–14 years but to a lesser extent.

**Figure 2.**
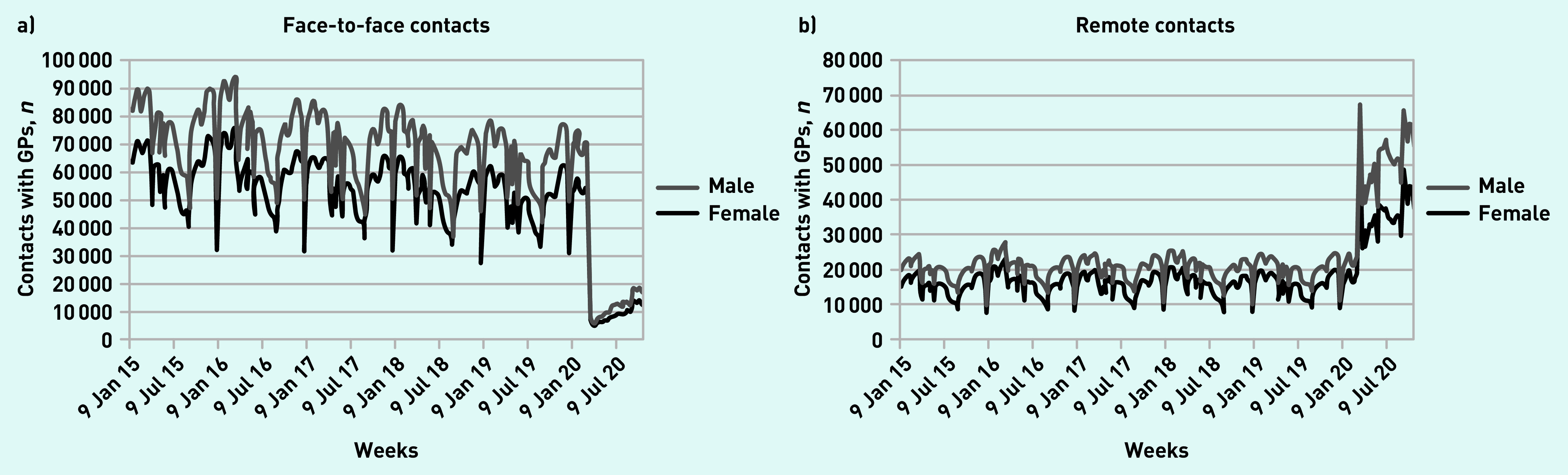
*Number of weekly contacts with GPs by sex. a) Number of face-to-face contacts for each week and b) number of remote contacts for each week.*

Changes in GP contacts were similar in males and females (Figure 2). Remote contacts in males increased by 119.5% (95% CI = 103.8 to 136.2) and by 127.6% in females (95% CI = 111.2 to 144.2) (data not shown). Face-to-face contacts fell by 88.4% in males (95% CI = 87.3 to 89.4) and by 88.2% in females (95% CI = 87.2 to 89.4).

**Figure 3.**
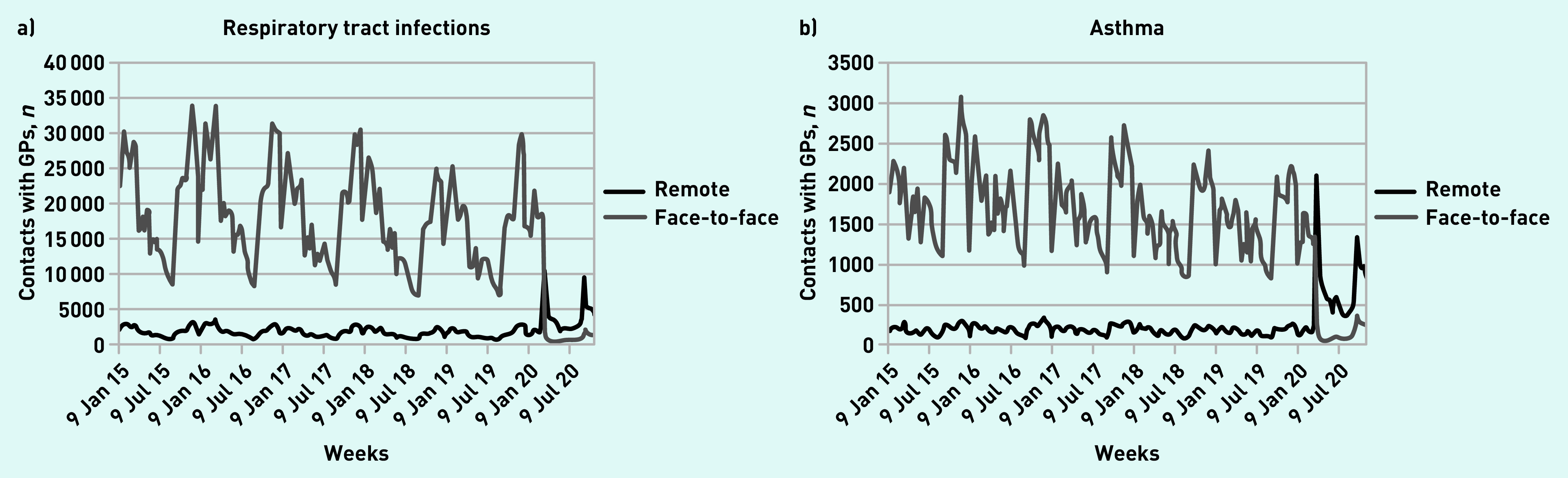
*Number of weekly face-to-face and remote contacts with GPs by selected respiratory illnesses. a) Respiratory tract infections and b) asthma.*

Contact patterns for respiratory illnesses and primary care sensitive conditions followed similar trends to the overall patterns, with a large drop in face-to-face contacts and a switch to remote contacts. Remote contacts for CYP for respiratory illnesses (respiratory tract infections and asthma) spiked initially at the start of the first lockdown, followed by another spike in remote contacts in September 2020 coinciding with schools reopening (Figure 3). Face-to-face contacts fell by 96.9% (95% CI = 96.0 to 97.5) for respiratory tract infections and 94.6% (95% CI = 92.7 to 95.9) for asthma (data not shown).

**Figure 4.**
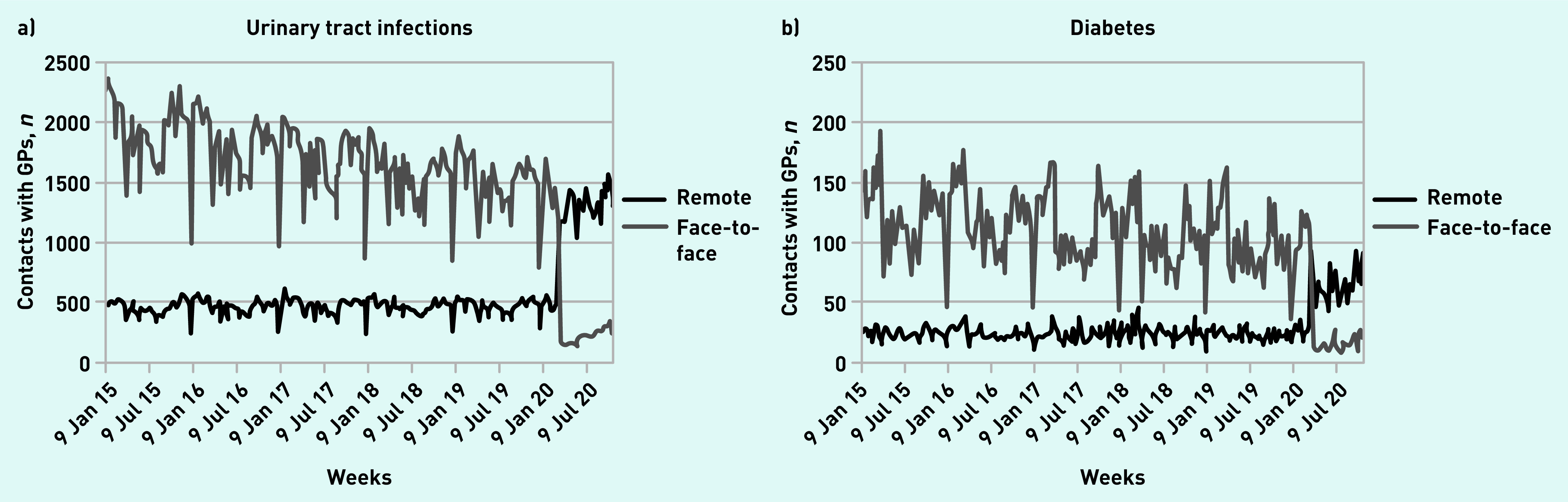
*Number of weekly face-to-face and remote contacts with GPs by selected primary care sensitive conditions. a) Urinary tract infections and b) diabetes.*

GP contacts for primary care sensitive conditions (urinary tract infections, appendicitis, diabetes, and epilepsy) shifted from face-to-face contacts to remote contacts during the lockdown period, but the drop in total contacts was only 30.9% (95% CI = 23.2 to 33.6) during the lockdown weeks compared with a fall of 74.0% (95% CI = 66.7 to 79.5) in total contacts for respiratory tract infections and asthma. Patterns for selected primary care sensitive conditions are shown in Figure 4, with the remaining conditions in Supplementary Figure S2.

## DISCUSSION

### Summary

CYP’s GP contacts fell 41% during the first lockdown of the COVID-19 pandemic. Face-to-face contacts fell 88% overall, with the greatest falls among children aged 1–14 years (>90%) and a slightly smaller drop for infants (77%). There was a shift to remote contacts in all age groups, but large increases in remote contacts for infants and young people aged 15–24 years mitigated total falls. Contacts for respiratory illnesses fell dramatically (74%) whereas contacts for primary care sensitive conditions had less of a drop overall (31%) because of the shift from face-to-face to remote.

### Strengths and limitations

To the authors’ knowledge, this is the first national report of children’s GP consulting patterns in the COVID-19 era. Its strengths include the large study population that is broadly representative of CYP in England and allows comparison with data from the previous 5 years. Therefore, it is highly unlikely the differences found in this study occurred by chance or sample selection effects, or could be explained by longer-term trends in healthcare activity.

Another strength of this study is that the baseline comparison averaged 5 years of data from 2015 to 2019. The mean from 2015 to 2019 is consistent and therefore a good comparator. In a sensitivity analysis comparing GP contacts from 2020 to the year 2019 only had a small impact on the main findings. However, there are some limitations to note in interpreting the findings of this study. In common with other large observational studies, data accuracy and completeness are variable. For example, administrative and clinical codes were used to differentiate face-to-face from remote contacts (see Supplementary Box S1). This may have resulted in some misclassification. In line with the specific aims of this study only contacts with GPs were looked at, and contacts with other providers such as practice nurses, pharmacists, or physician assistants whose roles have expanded in recent years were not examined.

### Comparison with existing literature

This study’s findings of falls in GP contacts with CYP are consistent with other reports of a drop in face-to-face contacts in adults and switch to remote contacts during the pandemic lockdowns of 2020.^27^^,^^28^ Unlike a study primarily in adults that suggested primary care contacts returned to pre-pandemic levels,^29^ this study found that by October 2020 CYP’s contacts had recovered but remained at around 18% below pre-pandemic levels.

Possible explanations for these findings include changing disease incidence, altered health-seeking behaviour, and changes to physician-consulting behaviour and services, all of which appear to have been affected by the pandemic.^30^ Social distancing measures and lockdown restrictions intended to reduce transmission of COVID-19 will also reduce other circulating viruses. Hence, infectious illnesses, which are among the most common reasons for children’s attendance in primary care, fell during the lockdowns,^31^ and an increase in incidence of infectious illness may also explain the spikes noted in this study in remote contacts at the start of the first wave of the pandemic in March 2020 and coinciding with schools reopening/restrictions easing in September 2020.^32^

Strong public messaging to ‘Stay at home, protect the NHS and save lives’ during lockdowns will inevitably have kept some patients away from health settings. Adherence to lockdown measures may have led to more management of minor childhood illnesses at home, and there is evidence from other countries that adults consulted GPs less frequently during the initial lockdown in 2020 for some minor conditions.^33^ Some parents expressed fear of exposure to COVID-19 from health settings^34^ whereas others reported incorrectly that GP practices were closed to face-to-face consultations.^35^^,^^36^ Misinformation about the availability of GP appointments and face-to-face consulting has been widely disseminated by social and news media.^37^

### Implications for research and practice

Children’s access to primary care has been falling in the UK and other countries for decades, accompanied by concomitant rises in use of urgent care including emergency department visits and hospital admissions.^10^^,^^11^ Thus pandemic disruptions have had a significant impact against a backdrop of existing pressures to provide GP contacts. The authors estimate that the falls of 41% resulted in 2 785 611 fewer GP contacts with CYP in England from March to June 2020 compared with previous years. However, the finding that GP contacts for respiratory illnesses fell drove a significant part of this decline, which was likely because of reduced transmission from containment measures. Emergency department attendances and child mortality for seasonal respiratory and gastrointestinal infection has reportedly fallen during lockdowns^38^ but is unlikely to be sustained as schools and educational settings reopen.

The findings of this study that remote contacts with young people more than doubled may indicate the shift to remote consulting by phone or video may have facilitated access to GPs for some younger people who often have excellent digital literacy and access.^39^ However, there may have also been difficulties in accessing remote care because of lack of space to speak privately and insufficient phone data or internet connectivity to take video calls.^40^ Access and utilisation challenges disproportionately affect CYP living in more deprived areas.^41^ Reassuringly, this study found that the total contacts for primary care sensitive conditions such as urinary tract infections, appendicitis, and epilepsy remained relatively high, and international reports confirm the incidence of these conditions changed little during this time.^42^ There is little evidence of a rise in serious illness in children during the lockdowns,^13^^,^^38^^,^^43^ suggesting that relatively few CYP experienced significant harm because of delays in seeking care.

The system-wide reorganisations of 2020 came after several years of primary care reforms. GPs have faced rising patient demand for appointments, an increasing workload because of a shift from hospital specialists to community care, and the demands of an ageing population with complex needs.^44^ Underinvestment in primary care to meet this rising workload has had an adverse impact on morale, with record numbers of GP principals leaving the profession, and has led to a declining workforce.

It is important to consider key lessons from the recent health system response.^45^ Although the current study has reported the health system was able to adapt and respond to short-term pandemic disruptions, further work is needed to understand the long-term impact of preventive primary care on CYP’s health.^46^

Remote and face-to-face consultations both have distinct advantages *.* Particularly at the start of the COVID-19 pandemic remote consultations offered opportunities for triage and provided practical solutions for GP contact with those shielding or self-isolating.^47^^,^^48^ As lockdown eased and consultation rates increased from July 2020, some GPs reported remote consulting became more time consuming, challenging, and fragmented.^48^ As the UK emerges from the winter of 2021–2022 with schools fully open, acute infection rates are rising and demand for acute appointments remains high. During the future anticipated peaks in infectious illnesses, such as respiratory syncytial virus, there may be a shortfall in primary care capacity for increased demand and need for face-to-face assessments to ensure safety and quality of children’s primary care.^49^ A picture is emerging whereby a hybrid model designed around patient preference is likely to replace traditional primary care.^50^ More work is needed to understand the optimal combination.^51^^,^^52^ The rebuild offers great opportunities to codesign primary care with CYP’s preferences.

The NHS Long Term Plan,^53^ published before the pandemic, has encouraged a shift towards integrated care systems to bring together NHS services, local authorities, and the voluntary sector for public health improvement.^54^ In addition to integration, the plan promises better prevention, workforce support, investment in digital infrastructure, and a better start to life for all CYP. It is essential that in this reorganisation, CYP are included in the plans for primary care reform.

In conclusion, during the first phases of the pandemic, CYP’s contact with GPs, particularly for face-to-face assessment, was lower than observed in adult populations.^27^ A major part of this shift may be ascribed to the change in prevalence of seasonal viral infections. Most contacts were held remotely but face-to-face consulting for infants was less disrupted and there was a major shift to remote contacts for primary care sensitive conditions mitigating overall falls. In reforming primary care services, the priority must be to consult stakeholders, including GPs and children and families, build safe digital and physical infrastructure, and support the primary care workforce to meet demand and to deliver better health outcomes for the whole population.

## Data

Additional unpublished data related to this study are not available. Access to CPRD data is only available to researchers with approved protocols via CPRD’s Research Data Governance Process. Code lists are available in the supplementary files (see Method section) and are shared without investigator support.
